# Effects of age and diet on broiler odor during a sustainable feed trial

**DOI:** 10.1016/j.psj.2026.107725

**Published:** 2026-09-04

**Authors:** Pascalle J.M. Deenekamp, Marleen Huisman, Flavia F. Schenone Castro, Joris Meurs, Simona M. Cristescu

**Affiliations:** Life Science Trace Detection Laboratory, Joris Meurs Radboud University, the Netherlands

**Keywords:** Poultry, Odor, Sustainable diet, Volatile organic compound, Mass spectrometry

## Abstract

Odor nuisance from industrial broiler farms is driven by complex mixtures of volatile organic compounds (VOCs), yet little is known about the longitudinal development of the VOC profile during the production cycle and how diet influences those odor profiles. This pilot study provided an initial characterization of the VOC profile of feces and litter from broiler chickens at three time points in the production cycle, for dietary interventions mimicking a transition to more sustainable low-opportunity cost feed (LCF) ingredients. Three diets were applied: a control (C), low starch (LS) and low protein and starch (LSP) diet. Non-target screening (NTS) using thermal desorption–gas chromatography–mass spectrometry (TD‑GC‑MS) was used to characterize the VOC profile, and 101 VOCs were putatively identified. Although biological variation between samples was high, VOC composition of litter and feces was still found to shift significantly over time. Various ubiquitous poultry-related odorants were found to drive this shift over time in all diets. Therefore, this work provides a first resource towards more effective, time‑specific odor mitigation strategies. On the final sampling day, 8 oxygenated VOCs, including several known livestock odorants, were found to be significantly different between diets. The LS diet showed decreased abundance for 6 out of 8 odorants, suggesting that a transition to sustainable feed materials has the potential to also positively influence odor emissions when diets are balanced for essential nutrients and amino acids.

## Highlights

• Non-targeted TD-GC-MS analysis annotated 100+ VOCs in broiler litter and feces.
• Dietary interventions reflect transition to low-opportunity-cost feed ingredients.
• Lower-starch diet lowers key odor-related VOCs.
• Key odorants shift over time, supporting targeted odor reduction strategies.

## Graphical abstract

**Figure:**
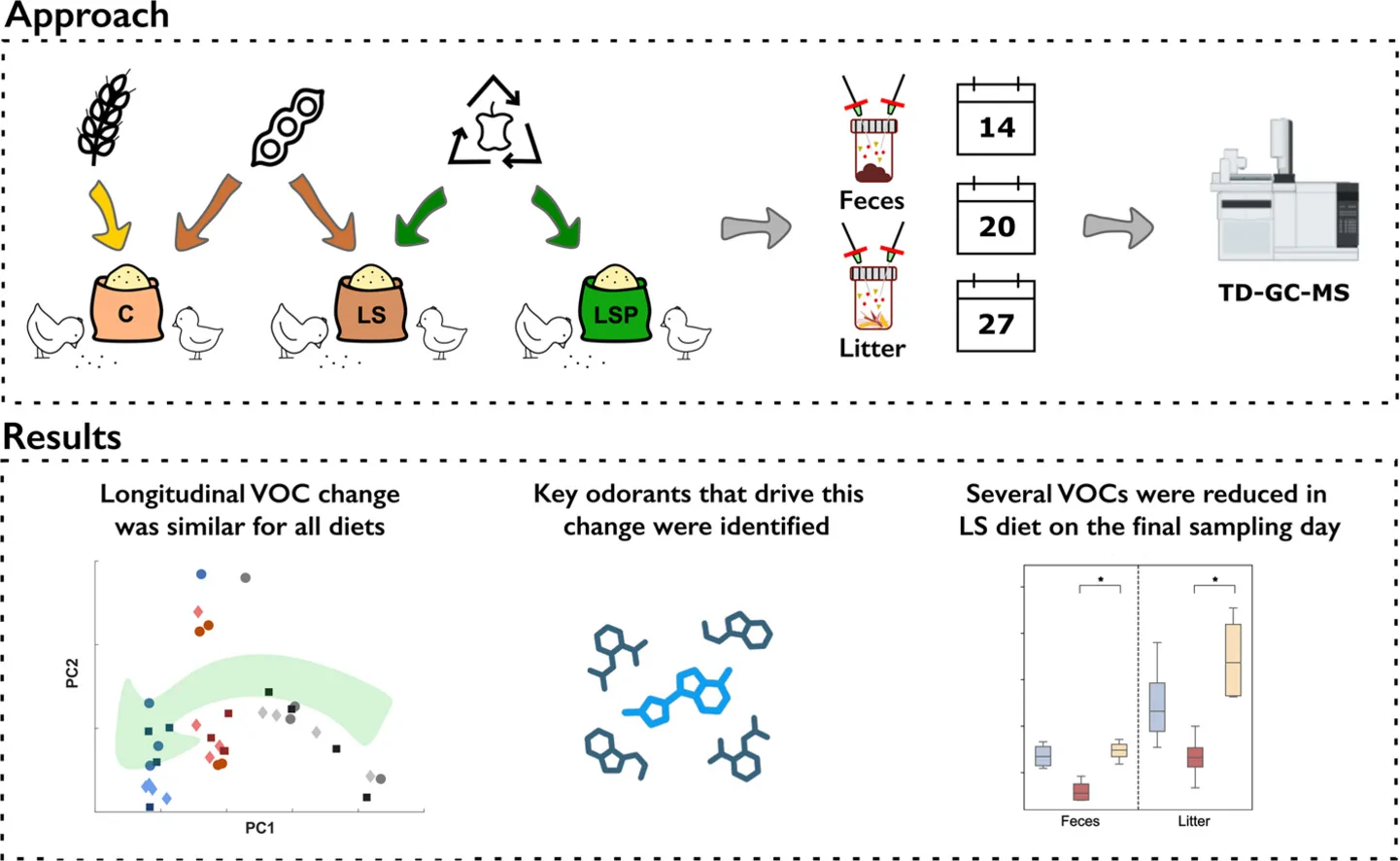

## Introduction

The livestock industry plays a fundamental role in providing the increasing global population with essential items such as food and clothing, yet its environmental impact is a key issue. Poultry accounts for more than half of the global meat production (FAO, 2023) and is one of the most efficient forms of meat production in terms of water use and greenhouse gas (GHG) emissions (Ramirez et al., 2021; Gržinić et al., 2023). However, broilers are commonly housed in concentrated animal farming operations, with stocking densities of up to 42 kg/m^2^ in the EU, corresponding to 15-20 broilers/m^2^ at the end of the cycle (Curea et al., 2023). Apart from animal health and welfare concerns, such farming operations also generate large amounts of waste material and emit dust, GHG, ammonia and odorous volatile organic compounds (VOCs) to the surrounding area (Bist et al., 2023). Malodorous compounds can have adverse effects on air quality and quality of life for the surrounding population, and reduction strategies mostly center on either treating exhaust air or mitigating odor production inside the farm (Gutarowska et al., 2014; Kalus et al., 2020; Zhu et al., 2023; Costello et al., 2024).

Apart from odor reduction, multiple other aspects of the poultry production industry present targets for improving sustainability. The largest environmental burden is caused by the production and transportation of feed ingredients, especially starch and protein, sourced mainly from cereal grains and South American soya, respectively (Leinonen and Kyriazakis, 2016; Mottet et al., 2017; Van Selm et al., 2022; Bikker and Jansman, 2023). Animal feed produced from low-opportunity cost (LCF) feed ingredients such as crop residues and food waste would have a substantially lower environmental impact due to enhanced efficiency of resource use, less deforestation and transportation emissions, and reduced competition between feed and food (Mottet et al., 2017; Van Zanten et al., 2019; Puente-Rodríguez et al., 2022; Van Selm et al., 2022; Bikker and Jansman, 2023). However, achieving similar energy and nutrient levels with LCF ingredients will likely increase the fat, sugar and fiber content of the feed (Bikker and Jansman, 2023).

Transitioning to LCF-based diets will also have implications for the odors produced during a broiler cycle. Odorous VOCs likely originate from microbial degradation of undigested food in the hindgut (Mackie et al., 1998; Le et al., 2008a; Qaisrani et al., 2015); therefore, dietary intervention may alter their production due to shifts in substrate and/or microbial gut population. Although reducing crude protein has been shown to result in lower flux of several odorous compounds (Chavez et al., 2004; Sharma et al., 2015, 2017a), the effect of starch reduction and fat and fiber supplementation on odor or VOC emission has not been previously studied for broiler chickens. Yet, two studies researched the effect of non-digestible oligosaccharide addition to diet (Yang et al., 2016; Zhu et al., 2020) and found mitigating effects on specific odorous compounds for some oligosaccharides, likely due to a shift in the broiler gut microbiota.

Until now, scant attention has been paid to how the full VOC profile develops over time during a broiler cycle. Throughout the growth cycle, broiler feed, metabolism and gut microflora change (Kers et al., 2018; Feye et al., 2020), affecting the odors they emit. In most odor-related studies, only a single time point in the cycle is studied; when temporal emission dynamics are reported, only specific VOCs are analyzed (Sharma et al., 2015, 2017a), GHG emissions are studied instead of VOCs (Miles et al., 2011), or more subjective methods using odor panelists are applied (Pillai et al., 2012; Huang and Guo, 2020). The sample matrix used for analysis also differs, as most researchers analyze litter or air samples, whereas fresh feces or excreta, likely important odor sources, are less represented (Murphy et al., 2014; Dunlop et al., 2016).

In this pilot study, we characterize the dynamics in the VOC profile of broiler feces and litter through non-target screening (NTS) using thermal desorption – gas chromatography – mass spectrometry (TD-GC-MS), both over time during the production cycle and with dietary changes related to the transition to LCF-based ingredients. By taking this step towards mapping a comprehensive VOC profile of broiler litter and feces at three time points and for three dietary treatments, we aim to contribute to the development of more effective odor mitigation strategies as well as analyze the potential impact on odor emissions when transitioning to more sustainable feed ingredients.

## Materials and methods

### Ethical approval

All animal procedures were carried out in accordance with the Dutch Experiments on Animals Act and applicable European legislation (Directive 2010/63/EU). The study protocol was authorized by the Central Authority for Scientific Procedures on Animals (CCD), the Netherlands, under project license AVD10800202418332 (issued to Utrecht University, The Netherlands). Animal housing and experimental execution took place at the animal research facilities of Wageningen University and Research (CARUS, Wageningen, The Netherlands) under project license AVD40100202010104.

### Experimental design for animal trial

This experiment spanned 28 days and applied 3 grower diets with varying levels of soya-based protein and cereal-based wheat. For the first 11 days, all chicks received the same commercial starter feed. From day 11 to day 28, the 3 dietary groups received different experimental grower feeds. 3 dietary treatments were applied: a control (**C**), low starch (**LS**) and low starch and protein (**LSP**) diet. Diets were formulated to be isoenergetic and with similar levels of standardized ileal digestible amino acids (Research Diet Services BV, Wijk bij Duurstede, the Netherlands). The composition and nutritional specifications of the starter diet and the 3 grower diets are shown in Table 1. Nutrient levels were not measured but calculated, and small discrepancies cannot be excluded. Enzymes (phytase (Axtra PHY®, Danisco, Denmark) and carbohydrase (RONOZYME® MultiGrain, DSM-Firmenich, Switzerland)), were added to the feed to improve digestibility, and a coccidiostat (Maxiban® 160, Elanco, USA) was used.

**Table 1:** Ingredients and chemical composition of dietary treatments (as-fed basis). For amino acids, standard ileal digestibility (SID) values are given. The starter feed was the same for all treatments, grower feeds are indicated as C, LS and LSP.

	Starter	C	LS	LSP
**Ingredients (%)**				
Corn	39.80	40.00	33.83	30.97
Soybean meal	23.44	19.16	24.06	10.08
Wheat	15.00	28.19	15.00	15.00
Wheat middling	7.71		6.18	19.55
Soy oil	2.50	1.42	3.76	4.63
Sunflower meal	2.00	1.00	2.20	1.00
Oat	2.00	1.50	5.00	5.00
Oat hull	2.00	2.00	6.00	6.00
Limestone fine	1.48	1.21	1.18	1.27
Peas	1.00	1.00	1.00	1.00
Monocalcium phosphate	0.56	0.23	0.14	0.17
Potato protein	0.50	2.69		3.01
Vitamin/mineral premix^1^	0.50	0.50	0.50	0.50
Sodium bicarbonate	0.20	0.14	0.15	0.28
Sodium chloride	0.20	0.21	0.21	0.12
L-lysine HCl	0.37	0.27	0.29	0.46
DL-methionine	0.31	0.24	0.26	0.30
L-threonine	0.14	0.07	0.11	0.16
L-arginine	0.11	0.08		0.24
L-valine	0.10	0.02	0.06	0.13
L-isoleucine (90%)				0.06
Coccidiostat (Maxiban 160)	0.06	0.06	0.06	0.06
Phytase (Axtra PHY)	0.02	0.01	0.01	0.01
Carbohydrase (RONOZYME)	0.01	0.01	0.01	0.01
**Total**	100	100	100	100
**Calculated chemical composition (g/kg)**				
Crude protein	197	190	190	170
Starch	388.4	450	360	360
Crude fat	57	44	69	79
Ash	54	43	49	47
Crude fiber	41	33	55	58
Sugars		35	40	34
Lysine	11.5	10.7	10.7	10.7
Methionine	5.7	5.1	5.2	5.4
Cysteine	84	7.8	7.8	7.8
Threonine	7.4	6.9	6.9	6.9
Tryptophan	2.1	2.0	2.1	1.8
Isoleucine	7.0	7.1	6.9	6.4
Arginine	12.3	11.1	11.1	11.1
Valine	8.9	8.2	8.2	8.2
ME_broilers_ (kcal/kg)	2850	2950	2950	2950

1 Provided per kilogram of diet: Vitamin A (retinyl acetate) - 10,000 IU, vitamin D_3_ (cholecalciferol) - 2,500 IU, vitamin E (dl-a-tocopherol) - 50 mg, vitamin K_3_ (menadione) - 2.3 mg, vitamin B_1_ (thiamin) - 2.0 mg, vitamin B_2_ (riboflavin) - 7.5 mg, vitamin B_6_ (pyridoxine HCl) - 3.5 mg, vitamin B_12_ (cyanocobalamin) - 20 μg, niacin - 35 mg, d-pantothenic acid - 12 mg, choline chloride - 460 mg, folic acid - 1.0 mg, biotin - 0.2 mg, Fe (FeSO_4_.H_2_O) - 80 mg, Cu (CuSO_4_.5H_2_O) - 12 mg, Mn (MnO) - 85 mg; Zn (ZnS0_4_.H_2_O) - 60 mg; I (KI) - 1.2 mg, Se (Na_2_SeO_3_) - 0.25 mg.

### Broiler management

240 male Ross 308 broilers were obtained as one-day-old chicks from a commercial hatchery and were randomly assigned to the treatment groups. Single-sex was used to limit gender-based variation. Each group in this experiment consisted of 4 replicate pens and 20 birds per replicate. The pens were evenly distributed over two identical rooms. The order of the treatment pens was randomized per room. Chickens had ad libitum access to feed and water via one feed pan and five drinking nipples per pen. Each pen consisted of a floor area of 2.0 m^2^ (2.0 m × 1.0 m) and contained 4 kg of bedding material (wood shavings) before the animals were divided over the pens. All chickens were housed in the research facility “Carus” of Wageningen University (Wageningen, the Netherlands). For the first 3 days, the lighting schedule was set to continuous lighting to encourage feeding, drinking and activity. After, a light-dark cycle was used with respective durations of 18 and 6 hours to promote a normal circadian rhythm. Temperature control was set to 34 °C one day before the broilers were placed in the pen and was gradually lowered to 21 °C at day 28. Both rooms were mechanically ventilated to ensure a proper climate. Supplementary document S1 provides detailed information on measured temperature and relative humidity in both rooms, as well as a schematic view of the pen setup and an image of a typical pen at D19.

### Sample collection and analysis

Fecal (total excreta) and litter samples were collected from each of the 12 pens that were part of this study on day 14, 20 and 27. Each of the four replicates per sample type and per condition reflects the physiological response of each 20-bird cohort. Fecal samples were collected in custom-designed fecal boxes consisting of stainless-steel trays (40 cm × 32 cm × 6.5 cm) equipped with a tailored plastic slatted floor (BirdSupply, Mariënheem, The Netherlands). The fecal boxes were placed in the pens of the experimental groups on day 13, 19, and 26 around 15.00 h. The excreted feces were manually removed from the boxes the day after around 10.00 h, avoiding contamination from feathers or feed, and pooled into plastic bags. The pooled fecal matter was manually homogenized, after which 1 g was weighed into 30-mL Sterilin™ containers. While fecal matter was collected, 1 – 3 g of litter from the pen floor next to the fecal box was collected in 30-mL Sterilin™ containers (Thermo Scientific, Bleiswijk, The Netherlands).

All Sterilin™ containers were baked-out for 3 days at 60°C in a Heratherm™ oven (Thermo Fisher, Amsterdam, The Netherlands) prior to use, to minimize any VOC artefacts originating from the container, and weighed. After sample collection, containers were stored on ice for transport. At the laboratory, the samples were stored at −20°C and analyzed within 1 week of collection. Sample weight was also noted at this moment.

Sampling of VOCs was done using the same setup and preconditioning protocol as described before (Deenekamp et al., 2025). Briefly, VOCs were collected from the sample container headspace onto preconditioned Biomonitoring sorbent tubes (Markes International, Bridgend, United Kingdom) by purging hydrocarbon-free air (50 mL/min; Sensor Sense B.V., Nijmegen, The Netherlands) for 15 minutes. The corresponding gas lines (1/8″ perfluoroalkoxyalkane) were fitted with 0.2-µm Minisart™ NML SFCA syringe filters (Sartorius, Fisher Scientific, Bleiswijk, The Netherlands) to prevent contamination of both the gas lines and the sorbent tubes. Prior to VOC collection, the headspace was purged for 1 minute with hydrocarbon-free air at a flow rate of 50 mL/min. Blank controls consisted of 4 empty containers per sampling day, processed under identical conditions. After VOC collection, all tubes were purge-dried for 5 minutes with N_2_ gas (100 mL/min).

Analyses of collected VOCs were carried out on a GC-MS QP2010 Ultra (Shimadzu, Kyoto, Japan) equipped with a TD-20 thermal desorption unit (Shimadzu, Kyoto, Japan). The desorption temperature was set to 260°C and sorbent tubes were desorbed for 8 min under a 60 mL/min-flow of helium (6.0 Scientific; Linde Gas, Dieren, The Netherlands). Desorbed VOCs were recollected on a Tenax TA focusing trap (Shimadzu, Kyoto, Japan) which was kept at –20°C. After, the focusing trap was rapidly heated to 250°C for 5 min to release the VOCs which were then transferred onto a CP-Sil 19 CF capillary column (length: 25 m, internal diameter: 0.25 mm, film thickness: 1.2 µm; Agilent Technologies, Amstelveen, The Netherlands) at a split ratio of 5:1. The carrier gas was helium and the flow was set to 1.0 mL/min. The column temperature was initially set at 35°C, held for 3 minutes, and increased to a final temperature of 250°C at a rate of 5°C/min. The final temperature was held for 5 min.

### Data analysis

Chromatograms were converted to netCDF format and analyzed with PARADISe v6.0.1 (Johnsen et al., 2017; Quintanilla-Casas et al., 2023). Default parameters were used for modelling. Deconvolution intervals and optimal model fit were manually selected. For putative identification of molecules, spectra were matched against the NIST23 Mass Spectral Library (Shimadzu, Kyoto, Japan) using match factor scores. Match factors are a measure for spectral similarity (ranging from 0 to 999). A minimum match factor of 800 was used as threshold for identification (Amirav et al., 2024) . The output from PARADISe was further processed using in-house MATLAB (R2023a, The Mathworks Inc., Natick, MA) and Python (v3.11, Python Software Foundation) scripts. Missing values were replaced with 1/5 of the lowest feature value (Das et al., 2022) and peak areas were normalized for sample weight. Prior to multivariate analyses, data were autoscaled.

Principal component analysis (PCA) and partial least squares-discriminant analysis (PLS-DA) were used to explore differences between samples taken on different days and dietary treatment groups. All PLS-DA models were validated by running 1,000 permutations for each model (Westerhuis et al., 2008). Variable importance in projection (VIP) scores greater than 1 were used to select VOCs for univariate statistical comparison. Univariate statistical analysis of the differences between samples collected on D14 and D27 for each diet and sample type was performed using a Mann-Whitney test with Benjamini-Hochberg post-hoc correction. Univariate statistical comparison of the peak area of selected VOCs from the final sampling day (D27) for the 3 dietary treatments was performed using a Kruskal-Wallis test. Dunn's test was used for pairwise comparison of the results. For all statistical analyses, a p-value of less than 0.05 was considered significant.

## Results and discussion

### VOC profiles of feces and litter samples over time and with diet

**Figure 1:**
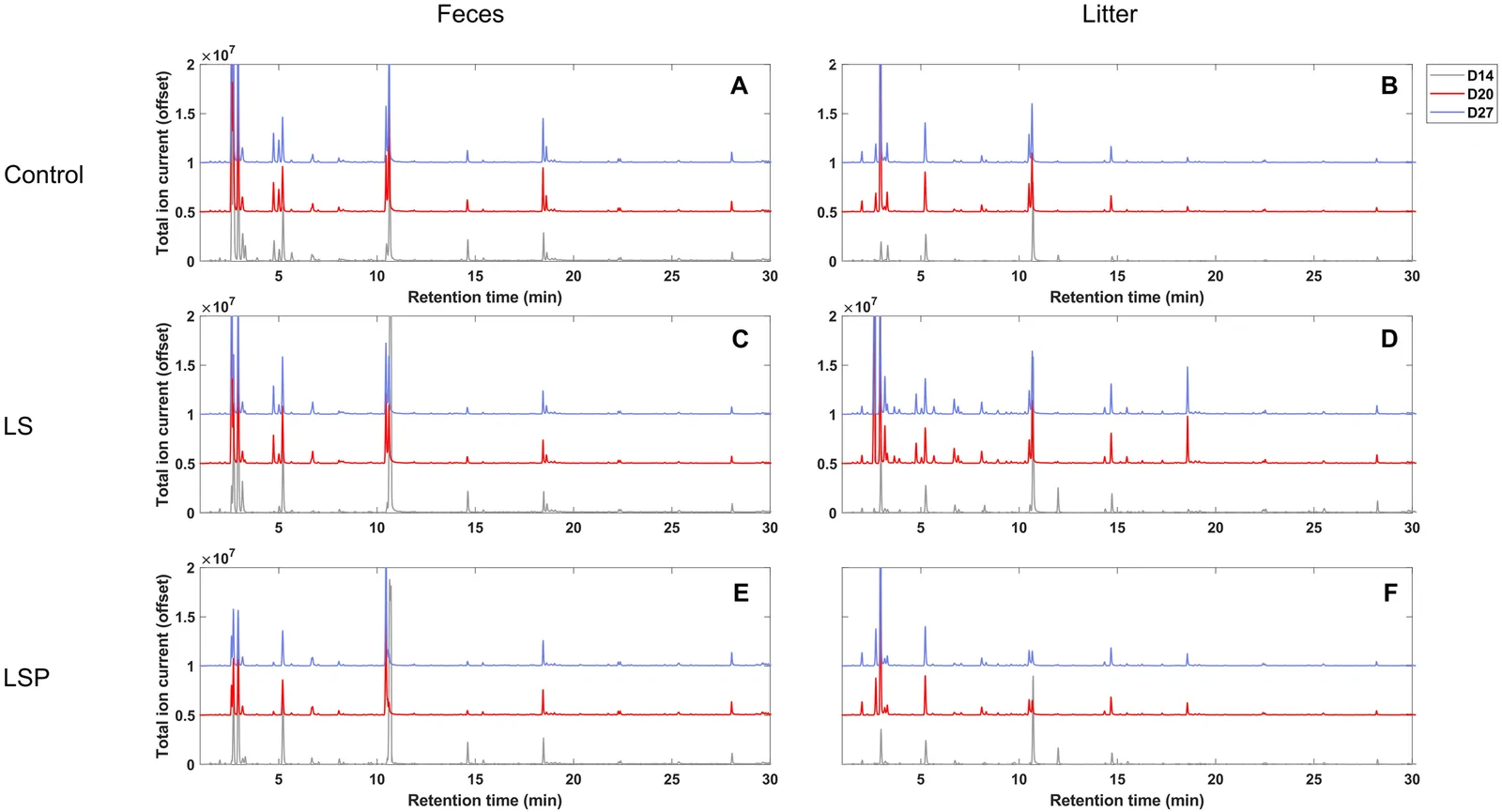
Median chromatograms for all sample types, days and dietary treatments. Panel A, C and E show the median chromatograms for feces headspace and diet C, LS and LSP respectively. Panel B, D and F show this data for litter headspace samples. The y-axis limits were fixed for visualization purposes.

Collected VOCs from feces and litter sample headspace across 3 sampling days and 3 dietary treatments were analyzed by TD-GC-MS. Figure 1 shows the median chromatograms per sample type (day-diet combinations). Over the displayed retention time range, distinct changes in abundance of numerous VOCs occur. This highlights the value of performing an unbiased NTS to gain an in-depth picture of time-related changes in the VOC profile.

The chromatograms were deconvoluted using PARADISe. In total, 126 peaks were found and 101 remained after manual removal of artefacts. Identities were assigned to the compounds by matching the mass spectra against the NIST23 mass spectral library. The remaining feature list was subjected to multivariate analysis to further explore time- and diet-related changes.

### VOC change during the cycle for all samples

**Figure 2:**
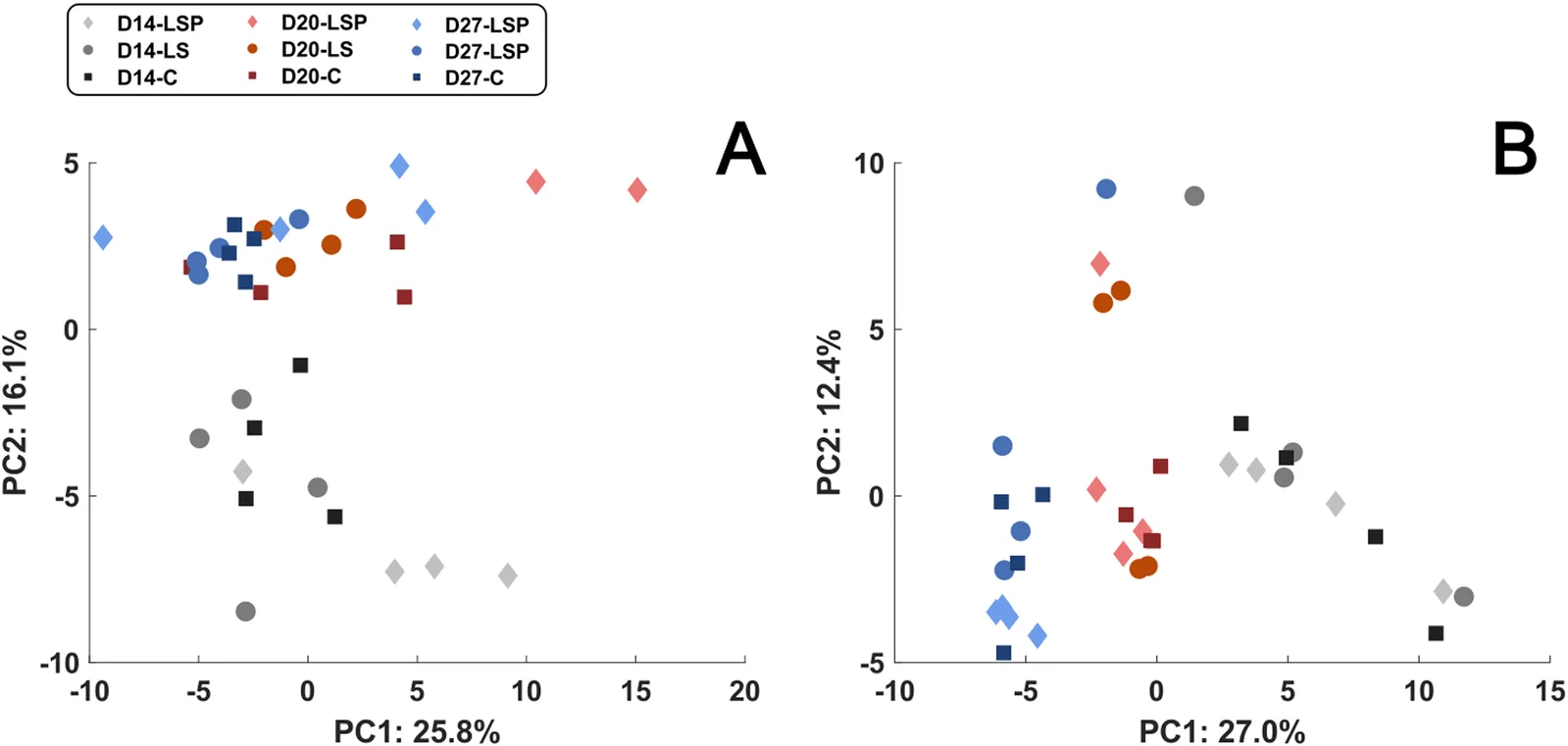
PCA scores plots for the two first principal components for A) feces and B) litter headspace VOCs.

For initial data visualization, PCA was used to explore variation and strong patterns in the dataset (Figure 2). Especially for the litter samples, progression, though not significant, (PERMANOVA: p = 0.5445 (time); p = 0.6933 (diet)) can be seen from D14 to D27 across the first principal component (Figure 2B). A progression can be also seen for the feces samples (Figure 2A), especially from D14 to D20 across the second principal component (PERMANOVA: p = 0.5544 (time); p = 0.0170 (diet)). Interestingly, the PERMANOVA result indicates there might be a change in fecal VOC profile driven by the type of diet. Due to technical issues with the TD-GC-MS, the data for 2 feces sample replicates of the LSP diet on D20 were lost, therefore only 2 data points are plotted. Variation between dietary sample replicates is high in this pilot study, especially for feces samples. This could be due to biological variation, as each chicken may have a very different health status or microbiome, or arise from sample processing, especially the manual homogenization, or storage and transport effects. Other possible contributions to variation are climate variations within and between rooms and broiler sex. Sex has been documented to have a strong effect on the bacterial population of the gut (Lumpkins et al., 2008) and therefore likely the odor profile. However, as single-sex broilers were used in this experiment, and climate control parameters in both rooms were identical and measured climate ranges were similar, these effects are likely limited compared to the sources of variation mentioned earlier.

Both the raw data and the PCA scores plots visually show that the VOC profile develops over time. As broilers have different nutritional requirements during their growth phases (Behnke and Beyer, 2002), this development over time is expected. Interestingly, the change seems visually similar regardless of dietary treatment, especially in the litter samples, which has not been previously reported to our knowledge. This provides a relevant insight for odor management strategies in broiler production facilities: odor mitigation strategies may need to be dynamic during a broiler production cycle, as specific VOCs may contribute to odor nuisance at specific time points.

**Figure 3:**
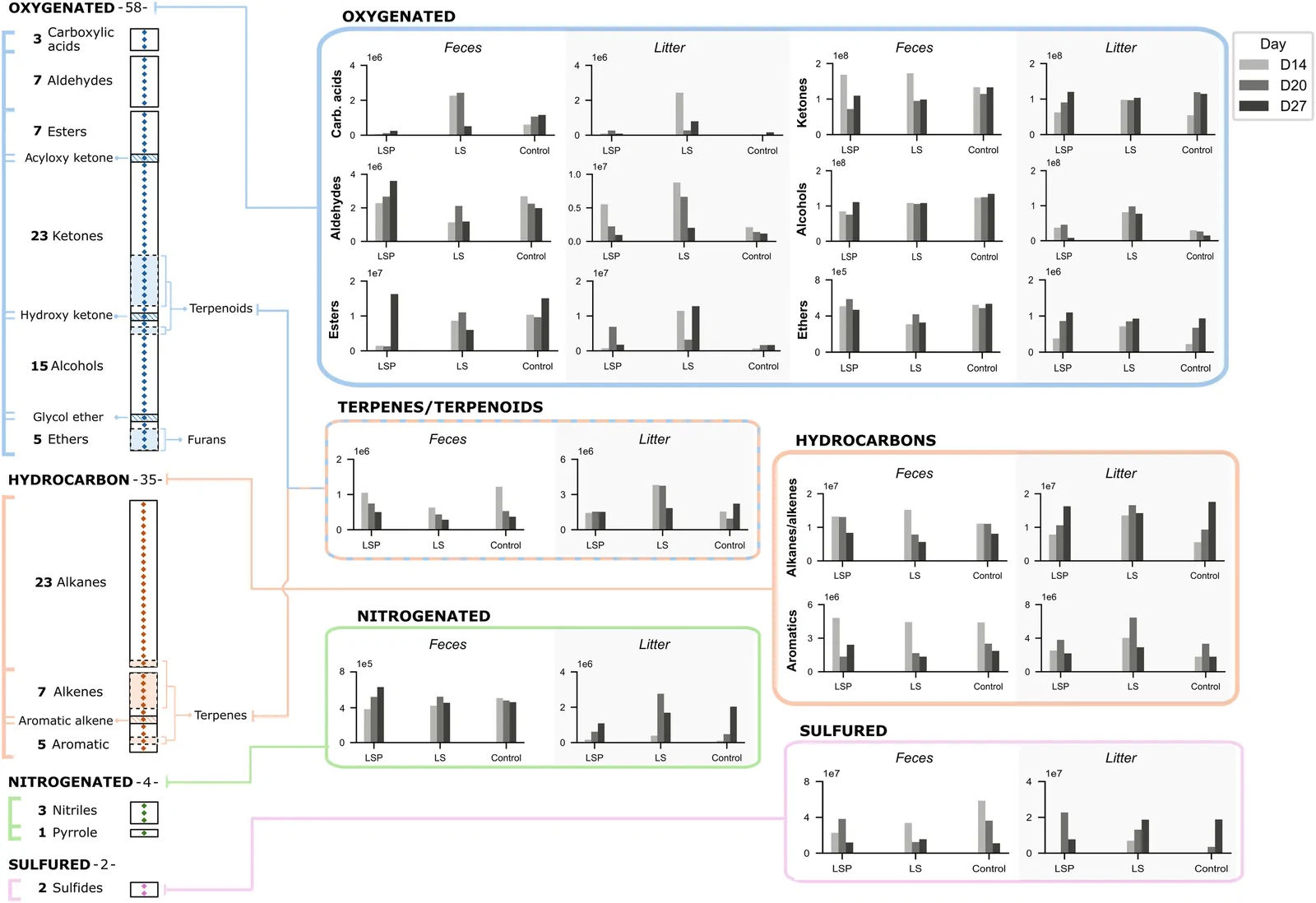
Chemical classes of detected VOCs. The figure on the left indicates the distribution of detected and annotated VOCs across the chemical classes. On the right, the total abundance (detected peak area) for each chemical class for the three sampling days is plotted for both fecal and litter samples.

To characterize this odor change, the annotated VOCs were grouped according to their chemical class (Figure 3). Terpenes and terpenoids were plotted as a separate chemical class, as the typical plant-related odors stemming from these secondary plant metabolites (Tetali, 2019) most likely originate from the bedding material in the litter. 4 molecules with multiple functional groups were included in both chemical classes.

In general, per sample type, the total abundance for many of the chemical classes develops similarly over time, supporting the observation from the PCA analysis. For instance, for non-terpenoid ketones, the change in abundance was similar across diets and sample type. In litter, aldehydes decrease from D14 to D27 in all treatments. As many aldehydes have floral or plant-like odor characteristics (Schade et al., 2001), this decrease is likely due to the original plant-like odors of the bedding material changing as the wood shavings age and mix with chicken excreta, water and spilled feed over time. Other oxygenated classes, such as alcohols, carboxylic acids and esters, were more variable in their behavior across dietary treatments and sample types, which could also be due to the high variation between dietary sample replicates as discussed in Figure 2.

In this analysis, mainly oxygenated compounds and aliphatic hydrocarbons were detected. Oxygenated compounds are often linked to unpleasant or characteristic odors (Trabue et al., 2010; Dunlop et al., 2016) and may be important targets for mitigation. Aliphatic and aromatic hydrocarbons can also cause odor nuisance (Meng et al., 2024).

It is established that the VOCs emitted during broiler production are a varied, complex mixture that is influenced by many factors, among which broiler age and diet (Casey et al., 2006; Trabue et al., 2010; Miles et al., 2011; Dunlop et al., 2013; Sharma et al., 2015). Our results corroborate this, showing a VOC profile with over 100 annotated compounds over a broad range of chemical classes. However, in previous odor studies, often a specific set of known odorants was monitored instead of a broader VOC profile. Multiple studies refer to research by Mackie et al. (1998) which lists major odorous VOCs such as volatile fatty acids and phenols (oxygenated), amines and indoles (nitrogenated), and sulfur-containing compounds. In our results, conversely, only a limited part of the total VOC profile was part of the sulfured and nitrogenated classes and phenols were not detected. Our results underline that in studies researching odor mitigation, it may be too simplistic to monitor only a known subset of VOCs with primarily N- and S-compounds, also because odor can arise from the interplay of different VOCs (Huang and Guo, 2020). However, as we do not detect several of the oft-reported odorants here, it would be beneficial to apply multiple collection methods to grasp the VOC profile more fully in future research. Only stainless-steel dual-bed sorbent tubes were used here for VOC capture, which could lead to bias for certain compounds (Kim and Kim, 2013). Further, the use of cellulose acetate filters to prevent dust and moisture from entering the sorbent tube, may also have retained specific VOCs.

### Key drivers of longitudinal VOC-profile shift

**Figure 4:**
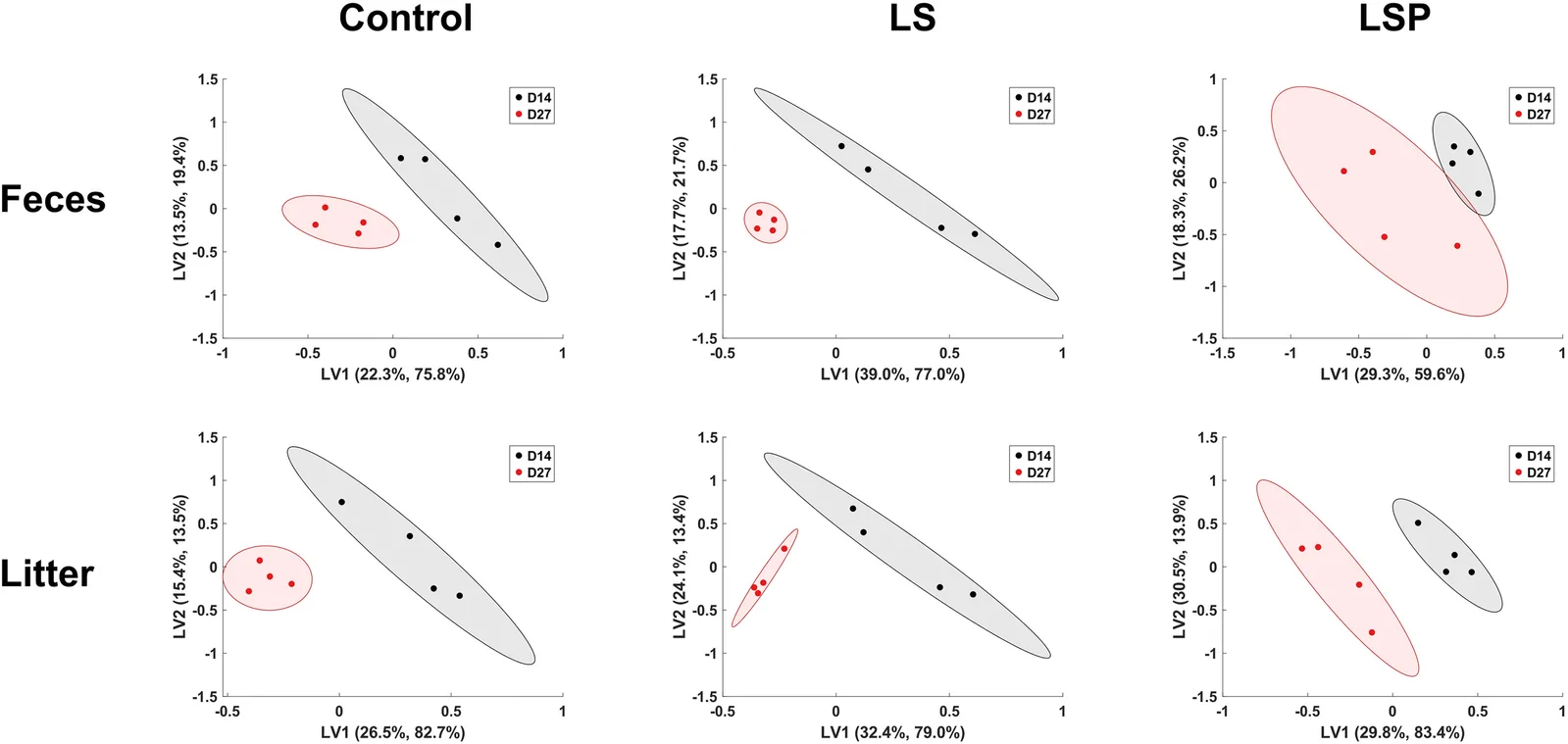
PLS-DA scores plots for the first and second latent variable for feces and litter for the three dietary treatments. D14 and D27 are compared for each.

To further elucidate the odor development over time, VOC profiles of the first and last sampling days were contrasted using PLS-DA, after which VIP scores were calculated to extract VOCs that could be influential in the odor development. Figure 4 shows the PLS-DA scores plots for all diets and samples for D14 and D27.

Figure 4 shows that the first and last days form distinct clusters for all diets and samples. Therefore, despite the often-high variation between replicates in this pilot study, the odor profile distinctly changes over time for both feces and litter samples, and across all diets, which matches the observations in the PCA scores plots (Figure 3). All PLS-DA models were validated using permutation testing. For all models, a p-value less than 0.05 was found indicating the models were not overfitted.

VIP scores were calculated to extract features that drive the difference in VOC profile between D14 and D27. Molecules with a VIP score > 1 for at least one of the diets were selected for further univariate analysis. This resulted in a list of 39 and 45 VOCs for feces and litter, respectively. A Mann-Whitney test with Benjamini-Hochberg post-hoc correction was used to identify VOCs that were significantly different between the 2 time points (p < 0.05), however, no VOCs met these criteria. This is likely due to the variation between replicates, which as discussed previously is high, likely due to biological variation and sampling, storage and transport effects.

**Figure 5:**
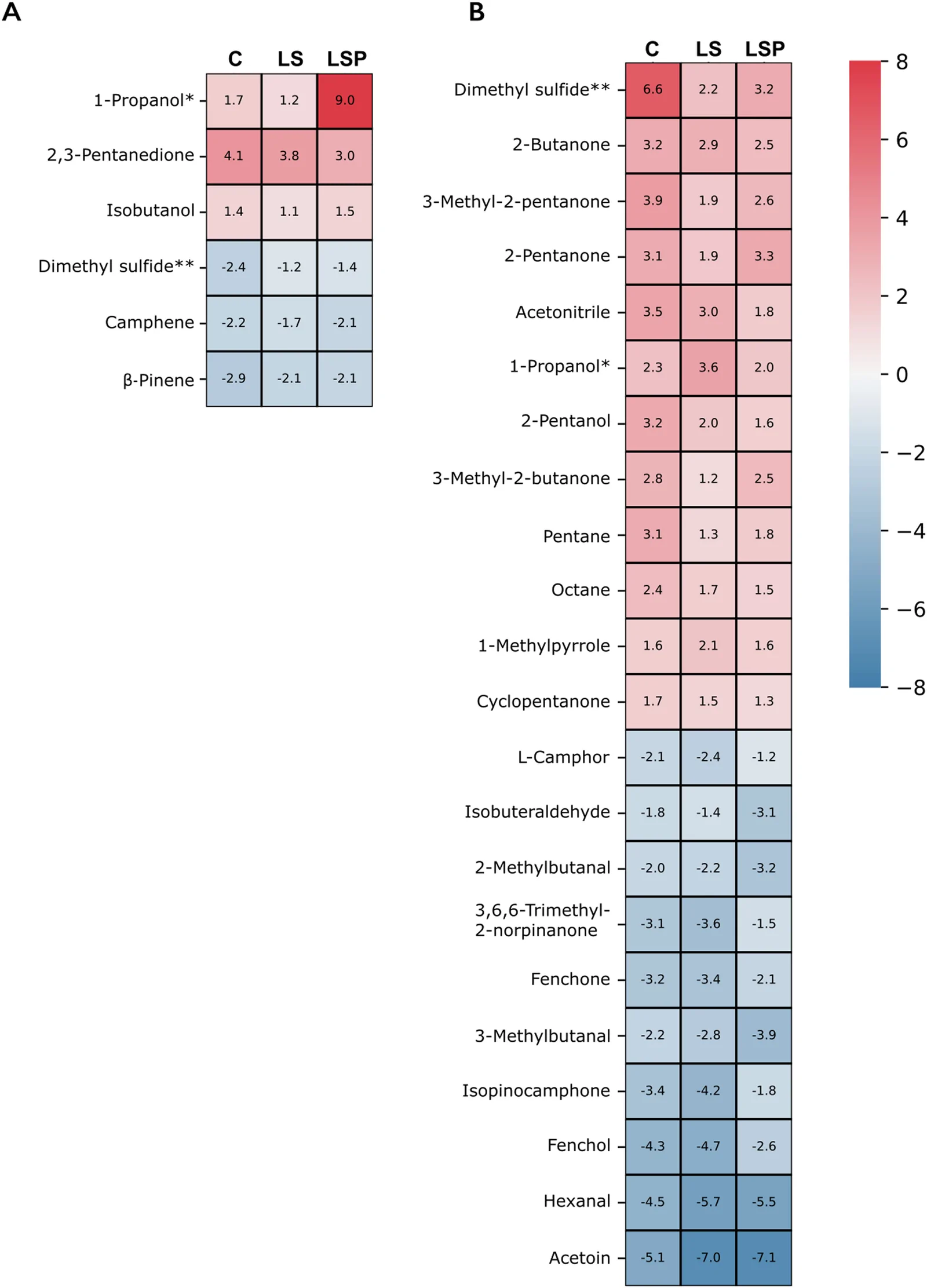
VIP>1 compounds for each diet with a log_2_fold change of at least 1 (increase) or −1 (decrease) between D14 and D27 in all diets for A) feces and B) litter samples.* VOC meets the log_2_fold criteria in both sample types and trends in the same direction in both. ** VOC meets the log_2_fold criteria in both sample types and trends in the opposite direction in the other sample type.

To narrow down which compounds are likely influential in the longitudinal change in odor profile that was observed regardless of diet, the log_2_ fold changes for this VIP>1 subset of molecules were calculated. Figure 5 shows the VOCs that had a positive or negative log_2_ fold change for all 3 diets between D14 and D27 of at least 1, corresponding to a 100% increase or decrease.

In total, 6 and 22 compounds change similarly in all diets for feces and litter samples, respectively. Although no single VOC but only the full VOC profile was significantly (p < 0.05) found to change over time in this exploratory study, these 6 and 22 single VOCs may offer interesting targets for the development of time-dependent odor mitigation strategies. As an example, acetoin, a key odorant from broiler farms (Le et al., 2005; Trabue et al., 2010; Murphy et al., 2014; Sharma et al., 2017b) trends downwards in litter in all diets (Figure 5B). Acetoin odor mitigation may be especially important early in the production cycle: it shows the strongest downward trend of all VOCs shown in Figure 5 (log_2_ values of 5 – 7.1, corresponding to 33 - 133 times less abundance on D27 compared to D14). Another key livestock odorant, 2-butanone, trends upwards in litter for all diets, indicating that odor mitigation strategies targeting this compound may become more important as the broilers grow.

Dimethyl sulfide is a sulfur-containing odorant with a strong contribution to odor offensiveness (Mackie et al., 1998), which trends in opposite directions in litter compared to feces, decreasing in feces over time but strongly increasing in litter. This observation underlines the importance of considering multiple sample types for odor sampling and mitigation, feces as well as litter.

Several terpenes and terpenoids, secondary plant metabolites (Tetali, 2019), trend down in both sample types. These VOCs likely originate from fresh bedding material at the beginning of the production cycle, and the decrease can be explained by the degradation of the material over time. Another possibly plant-derived VOC is 2,3-pentanedione, which increases strongly over time in fecal sample. It is a flavor compound that is prevalent in a wide variety of plant materials (Wishart et al., 2022) and could originate from plant-based feed ingredients. Although not reported before in earlier poultry-related VOC research, 2,3-pentanedione has been detected in the air of swine operations (Schiffman et al., 2001).

Interestingly, 1-propanol, which trends strongly upward in both sample types and exhibited the highest overall log_2_fold increase in feces in Figure 5, is little reported on in previous studies. It may contribute to pungent odors near the end of the cycle. The influence of this VOC on odor should be further investigated.

It is warranted to evaluate broiler odor nuisance at multiple time points during the cycle at commercial farming operations, as this semi-field research shows that key odorants evolve over time. Ideally, odor emission should be mapped every 1-2 weeks during the cycle to capture shifts. As some odorants develop differently across sample types, multiple sample types should be analyzed. The abundance of lesser-studied odorants was found to evolve strongly over time in this research, supporting that a broad VOC profile should be considered when evaluating odorous emissions.

Research shows that several strategies are available for odor mitigation. To highlight a few that are relevant to the VOCs found to shift in this study, both 2-butanone and 1-propanol emission, which increased over time in this study, was found to decrease upon dietary treatment with probiotics (Chang and Chen, 2003). Adding probiotics to the diet may therefore be most effective in the grower and finisher diets. Litter treatment may also be effective: zeolite litter treatment (Turan et al., 2009) was also found to mitigate 1-propanol emissions and general odor emission rates were found to decrease in a study by Costello et al. (2024) upon addition of lignite to broiler litter. Of course, for practical implementation, the economic feasibility of such odor monitoring and odor mitigation strategies should be evaluated.

### Influence of dietary treatment on VOC profile

We showed that odors change during a broiler production cycle and that these changes are, in general, similar regardless of diet. Although the effect of age was visually clear in both sample types from the exploratory PCA (Figure 2), PERMANOVA analysis indicated that diet type may also drive a change in VOC profile. This is supported by literature: according to multiple studies, dietary treatment has a strong influence on livestock odor (Chavez et al., 2004; Le et al., 2005; Willig et al., 2005; Pillai et al., 2012; Sharma et al., 2015; Yang et al., 2016; Zhu et al., 2020).

**Figure 6:**
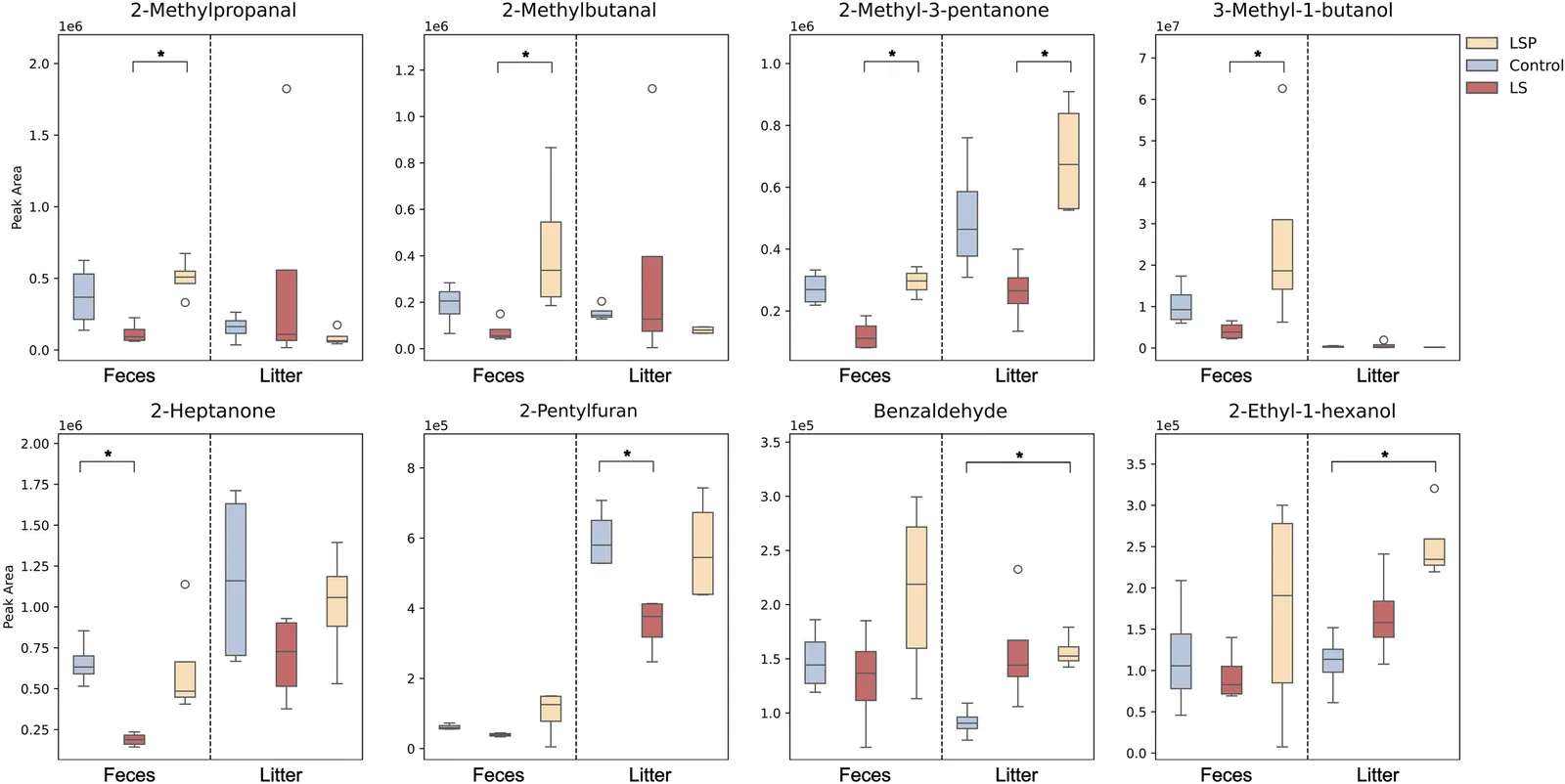
VOCs that were significantly different between diets on D27.

To assess the effect of the dietary treatment on VOC profile, the subset of VOCs (VIP>1) was compared between diets for the last measuring day only (D27) using Kruskal-Wallis test followed by Dunn’s test. This resulted in 5 and 4 molecules that were significantly different in at least one diet for feces and litter, respectively. The abundance of these VOCs is shown in Figure 6. Two of these VOCs, 2-methylbutanal and 2-methyl-3-pentanone, were also found to drive the longitudinal VOC profile shift in litter (Figure 5). Although they evolve similarly across time, their formation process also seems to be influenced by diet.

The 8 VOCs shown in Figure 6 have been previously linked to livestock and feces (Schiffman et al., 2001; Garner et al., 2007; Dunlop et al., 2013; Kaikiti et al., 2021). 3-Methyl-1-butanol, 2-methylpropanal and 2-methylbutanal were identified in other research as key odorants in poultry farms (Murphy et al., 2014; Sharma et al., 2015). 2-Methyl-3-pentanone, 2-heptanone and 2-ethyl-1-hexanol were previously reported in other livestock odor nuisance studies (Schiffman et al., 2001; Kaikiti et al., 2021). 4 out of these 5 odorants (all except 2-ethyl-1-hexanol) are decreased in abundance in the LS diet. Transitioning to more sustainable lower-starch diets with less cereal grains may therefore have positive impact on odor mitigation as well.

Research has established that odorous compounds from poultry are often a result of microbial fermentation of undigested proteins and carbohydrates in the hindgut and that dietary changes may result in odor change from change in substrate or microbiome shift (Mackie et al., 1998; Willig et al., 2005; Le et al., 2008b; Qaisrani et al., 2015). Previous studies of varying crude protein (CP) levels in diets often report an increase in odor with increased protein levels (Chavez et al., 2004; Sharma et al., 2015, 2017a). Interestingly, our findings do not support this: none of the VOCs in Figure 6 display lower abundance for the LSP diet. In all but 2 cases, the abundance was lowest for the LS diet. In both other cases (benzaldehyde and 2-ethyl-1-hexanol in litter), the abundance was significantly lower in the Control diet. The CP fraction alone, therefore, may not be the most important factor in odor production. Microbial fermentation of amino acids is linked to carbohydrate availability: when microbes do not have enough fermentable carbohydrates (FC) available, they may use protein as an energy source, leading to more odor-offensive compounds produced (Willig et al., 2005). Le et al. (2008b) found that odor emission was increased in porcine manure when CP levels were high relative to FC levels, while increasing FC levels at similar CP levels decreased odor emission. If microbes do not have a balanced ratio of FC and CP available, amino acids can be fermented, resulting in key odorous compounds. The results in Figure 6 support this: several VOCs that are reported to stem from amino acid degradation, 3-methyl-1-butanol, 2-methylpropanal and 2-methylbutanal (Marilley and Casey, 2004), were lowest in abundance in the LS diet. The LS diet may therefore be the most balanced of the 3 treatments in terms of necessary amino acids versus FC, resulting in lower abundance of odorous compounds.

The diets that mimicked a transition to LCF-based ingredients, LS and LSP, had a much higher fiber fraction than the C diet (C:33%, LS:55%, LSP:58%, see Table 1), but in terms of VOC abundance in Figure 6, the LSP and C diets are more similar to each other than to the LS diet. A high fiber fraction may lead to higher CP in the hindgut and more odorous VOCs, as dietary protein ‘locked’ in the fiber fraction may only become available after fermentation by the gut microbes (Bikker and Jansman, 2023). Pre-fermented feed additives resulted in lower production of various indoles in a study by Zhu et al. (2023), which corroborates this. Here, however, the almost doubled fiber fraction in LSP versus C did not result in significantly different VOCs. LCF-based diets may have higher fiber contents due to the use of alternative feed ingredients, but the potential effect on odor is unclear and likely dependent on the physio-chemical properties of the fibrous ingredients. Studies in pigs and poultry have shown that a higher fiber fraction may lead to more short-chain fatty acid production, with positive effects on gut functioning, but can also lead to dysbacteriosis and growth of pathogens (Jha et al., 2019).

### Study limitations

Several limitations of this pilot-like study should be acknowledged. First, the work was conducted under semi-field conditions and therefore may not fully represent the complexity of large-scale commercial poultry operations. Also, this study did not account for possible adverse effects of LCF-based diets on broiler growth, which could negatively influence VOC emissions per kilogram of produced meat.

In addition, a relatively low number of replicates was used per condition (n = 4) and variation between replicate samples was relatively high. Each replicate reflects the average physiological response of the entire 20-bird cohort, providing a robust estimate of group-level effects. The group-level approach does show high variability as it may reflect inherent biological differences between the pooled feces of 20 animals. Variability could also arise from sample handling and processing steps, such as the manual homogenization. A clearer understanding of the relative contribution of these sources of variation, such as analyzing additional sample replicates per pen to map within-pen variation, would strengthen interpretation of the results.

The sampling approach may also introduce analytical bias. Previous studies have noted that sorbent tubes can be suboptimal for capturing a broad range of VOCs, particularly when single-bed sorbents are used, and housing material may also have an influence (Woolfenden, 1997; Trabue et al., 2010; Kim and Kim, 2013). The dual-bed sorbent tubes applied here allow adsorption of a wider range of VOCs (C2-C30). However, selective adsorption may still occur, potentially leading to over- or underrepresentation of certain compounds or compound classes. Ideally, a combination of sampling techniques would be used to obtain a more comprehensive VOC profile. Further, cellulose acetate (CA) filters were used to trap dust and moisture, which may have selectively retained some previously reported odorous VOCs, such as nitrogenated compounds (e.g. indole). In complementary PTR-ToF-MS measurements performed with and without CA filters using an indole reference standard, we found that the signal intensity was reduced when a CA filter was attached (data not shown). This may help to explain our limited detection of nitrogenated and sulfurous compounds (only 6 detected VOCs were putatively identified to belong to these classes, as shown in Figure 3).

As we used a non-target screening approach, we only achieved identifications at Metabolomics Standard Initiative (MSI) level 2 (Sumner et al., 2007). Our main aim was to explore whether shifts occur in odor profile during a broiler cycle and what VOCs potentially play a role in these processes. To achieve the highest level of identification (MSI level 1), future studies should focus on a targeted analysis approach in which a (sub)set of VOCs reported here can be monitored and validated using authentic reference standards.

In conclusion, in this pilot study, multivariate analysis showed a strong longitudinal shift in VOC emission profiles from broiler feces and litter, outweighing the effect of dietary treatment. Several odorants that are often reported for broilers, such as acetoin and 2-butanone, changed markedly over time in all diets. These initial findings imply that effective odor-mitigation strategies may need to be time-dependent, tailored to stages in the production cycle.

In terms of the effect of diet on odor, the LS diet, with reduced dietary starch from cereal-based grain sources, was found to mitigate emission of several VOCs related to livestock odor nuisance on the final sampling day. Although protein reduction is often linked to odor mitigation in previous research, the LSP diet, with a combined reduction of soya-based protein and cereal-based starch, did not produce the same effect. From these explorative findings, protein reduction alone does not seem a decisive factor in diet-based odor control. For a more sustainable future of the broiler production industry, it is promising that our results show that replacing soya- and cereal-grain-based feed ingredients with LCF may not have adverse effects on emissions if diets are balanced for essential nutrients and amino acids. As this study was pilot-like in nature and conducted under semi-field conditions, future studies should validate these findings at field-scale and include further optimization of analytical procedures. Also, performance data and microbiome analysis could be integrated.

In short, although these findings are exploratory and study limitations should be addressed in a more comprehensive trial, our findings contribute to a better understanding of the VOCs that make up the odor of broiler waste materials and how these may change over time and due to dietary intervention. This work can provide a resource useful for the development of more effective, time-dependent odor mitigation strategies, and generates new insight on the potential odor effects of improving broiler husbandry sustainability through feed ingredient change.

## Author Contribution

Pascalle J.M. Deenekamp contributed to the conception and design of the study, methodology, investigation, data analysis and curation, creating visualizations, and preparation of the original manuscript draft. Marleen Huisman contributed to the methodology, investigation, data analysis, and preparation of the original manuscript draft. Flavia F. Schenone Castro contributed to the investigation, data analysis and curation and preparation of the original manuscript draft. Joris Meurs contributed to the funding acquisition, conception and design of the study, supervision, investigation, methodology and preparation of the original manuscript draft. Simona M. Cristescu contributed to the funding acquisition, supervision, methodology, and preparation of the original manuscript. All authors contributed substantially to the study, critically evaluated the manuscript, and have reviewed and explicitly approved its final version. Each author accepts personal and public responsibility for the integrity of the work and is committed to ensuring that any concerns regarding the accuracy or reliability of any aspect of the research are properly examined and resolved.

## Disclosures

The authors declare that they have no known competing financial interests or personal relationships that could have appeared to influence the work reported in this paper.

## Data Availability

The raw/processed data required to reproduce the above findings are available to download from the Radboud Data Repository:https://doi.org/10.34973/v00c-w866
