# Supplementary material for: Effects of age and diet on broiler odor during a sustainable feed trial
Source: Poult Sci. 2026 Sep 4;105(12):107725. doi: 10.1016/j.psj.2026.107725 (PMC13594904; doi:10.1016/j.psj.2026.107725)
Supplement: Supplementary file 1 [file mmc1.docx]

Supplementary info S1: Broiler Management Additional Information

Table 1:Temperature (T) and relative humidity (RH) target settings and measured ranges over the course of the experiment in room 1 and room 2.

| Day | T setting (°C) | Measured T range room 1 (°C) | Measured T range room 2 (°C) | RH target range (%) | RH room 1 (%) | RH room 2 (%) |
| --- | --- | --- | --- | --- | --- | --- |
| 0-1 | 34 | 33.9-34.9 | 33.9-35.0 | 50-60 | 52.3-57.2 | 52.1-56.9 |
| 2 | 33 | 30.9-33.4 | 30.4-34.0 | 50-60 | 56.5 – 62.1 | 52.5-61.7 |
| 3 | 31 | 31.2-33.1 | 31.0-32.3 | 50-60 | 57.0-60.0 | 56.8-61.2 |
| 4-6 | 29 | 28.1-32.1 | 26.4-31.7 | 50-60 | 51.3-61.4 | 51.2-58.2 |
| 7-9 | 28 | 26.4-28.9 | 26.7-28.8 | 40-60 | 48.6-53.8 | 46.8-60.7 |
| 10-12 | 27 | 24.5-28.3 | 24.5-28.8 | 40-70 | 48.7-53.2 | 49.0-63.0 |
| 13-15 | 26 | 24.4-26.7 | 24.3-26.8 | 40-70 | 43.8-68.5 | 46.6-69.1 |
| 16-17 | 25 | 24.8-26.2 | 24.8-26.1 | 40-70 | 57.6-68.0 | 57.2-68.2 |
| 18-20 | 24 | 24.6-26.4 | 24.4-25.6 | 40-70 | 52.4-64.5 | 46.1-68.5 |
| 21-22 | 23 | 23.2-25.6 | 23.2-25.3 | 40-70 | 52.4-65.4 | 57.4-71.4 |
| 24-26 | 22 | 22.2-24.2 | 21.9-24.0 | 40-70 | 51.1-67.0 | 53.7-68.2 |
| 27 | 21 | 22.1-23.5 | 21.7-24.1 | 40-70 | 47.5-60.0 | 50.0-64.0 |
| 28 | 20 | 23.0-24.7 | 21.8-24.1 | 40-70 | 46.7-61.4 | 45.5-63.6 |

Figure 1: Schematic view of the distribution of the sampled pens over the two rooms. Dietary treatments LSP, LS and C are indicated and were distributed randomly over the two rooms. The unmarked pens were occupied with broilers of the same age, sex and breed, but were not part of this study.

Figure S2:A photograph of one of the pens at broiler age of 19 days. The feeding pan and 4 of the five drinking nipples are visible.
